# Understanding the Notch Signaling Pathway in Acute Myeloid Leukemia Stem Cells: From Hematopoiesis to Neoplasia

**DOI:** 10.3390/cancers14061459

**Published:** 2022-03-12

**Authors:** Daniel Láinez-González, Juana Serrano-López, Juan Manuel Alonso-Dominguez

**Affiliations:** 1 Experimental Hematology, Instituto de Investigación Sanitaria Fundación Jiménez Díaz, 28040 Madrid, Spain; daniel.lainez@fjd.es (D.L.-G.); juana.serrano@quironsalud.es (J.S.-L.); 2 Hematology Department, Hospital Universitario Fundación Jiménez Díaz, 28040 Madrid, Spain

**Keywords:** Notch, quiescence, AML, LSCs, crosstalk

## Abstract

**Simple Summary:**

We review the state of the art of knowledge regarding the Notch signaling pathway to shed light on the role that this pathway has in hematopoiesis and hematological neoplasia, focusing on acute myeloid leukemia.

**Abstract:**

The Notch signaling pathway is fundamental to early fetal development, but its role in acute myeloid leukemia is still unclear. It is important to elucidate the function that contains Notch, not only in acute myeloid leukemia, but in leukemic stem cells (LSCs). LSCs seem to be the principal cause of patient relapse. This population is in a quiescent state. Signaling pathways that govern this process must be understood to increase the chemosensitivity of this compartment. In this review, we focus on the conserved Notch signaling pathway, and its repercussions in hematopoiesis and hematological neoplasia. We found in the literature both visions regarding Notch activity in acute myeloid leukemia. On one hand, the activation of Notch leads to cell proliferation, on the other hand, the activation of Notch leads to cell cycle arrest. This dilemma requires further experiments to be answered, in order to understand the role of Notch not only in acute myeloid leukemia, but especially in LSCs.

## 1. Introduction

Notch has an important role in several functions related to embryogenesis and cell fate in adult tissues. Indeed, Notch signaling has been extensively linked to both processes of normal and malignant stem cell (SC) self-renewal [1,2]. For this reason, it is important to comprehend its implications not only in its physiological form, but also in its aberrant variety. Our objective with this review is to summarize the available information about the hematological role of Notch, mainly in the regulation of the leukemic stem cells (LSCs) of acute myeloid leukemia (AML). In this paper, we also examine the interdependence of Notch signaling with Hedgehog (Hh) and Wnt. These 3 pathways are connected during embryo development, but also in SC regulation and differentiation in different tissues.

For this purpose, we employ the PubMed database by using the following terms: Notch, quiescence, LSCs, leukemic stem cell, acute myeloid leukemia, crosstalk, Hedgehog and Wnt.

## 2. Notch Signaling Pathway

Two important works described the Notch signaling pathway at the beginning of 20th century. In 1914, Dexter analyzed variations in Notch-mutated phenotypes of the fly wing of *Drosophila melanogaster* [3]. Some years later, in 1917, Morgan identified the mutant alleles [4]. After these two milestones, almost 70 years passed before Wharton presented the primary structure of the Notch transcript [5]. To date, Notch has been studied in different species, concluding that this signaling pathway has been conserved from numerous metazoan organisms, such as *Caenorhabditis elegans* to *Homo sapiens* [6,7,8].

The ligand receptors in Notch are a single-pass type 1 transmembrane protein called Delta and Serrate, in D. melanogaster; otherwise, other species have only one receptor called LAG1, as in C. elegans. Nevertheless, the collective term for these proteins is DSL. On the other hand, in mammals, Notch ligands are classified into two groups: Delta-like (DLL1, DLL3, DLL4) and Serrate (Jagged)-like (JAG1, JAG2) [9,10].

If we focus on the receptors of Notch, mammals, such as mice and humans, present four Notch receptors (NOTCH1–4). Although these proteins are similar among them, they have some differences; NOTCH1 and NOTCH2 receptors have 36 EGF-like repeats in their ectodomain. NOTCH1 contains a strong transactivation domain (TAD), whereas NOTCH2 has a weak TAD. On the contrary, NOTCH3 and NOTCH4 have no TAD in the endodomain and they are formed by 34 and 29 EGF-like repeats, respectively [11]. The composition of NOTCH proteins is fundamental for its canonical activation. These proteins have two domains: the Notch extracellular domain (NECD) and the Notch intracellular domain (NICD) [10].

**Figure 1:**
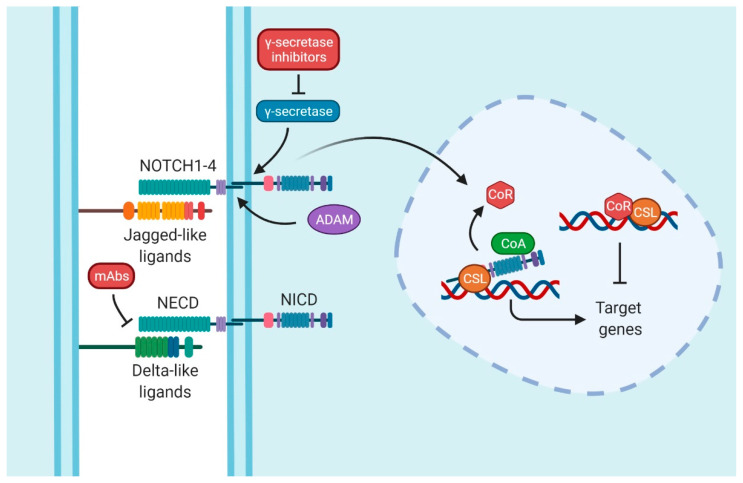
Notch signaling pathway. The interaction between Delta-like or Jagged-like ligands and NOTCH entails the activation of the signaling pathway. Whereas the extracellular domain (NECD) is split by a disintegrin and metalloproteinase (ADAM), the intracellular domain (NICD) is released by γ-secretase. Therefore, NICD translocates into the nucleus, displacing the co-repressor proteins (CoR) and promoting the target genes with the assistance of co-activators proteins (CoA) and CSL (CBF1/RBPJ-κ, suppressor of Hairless and LAG1). The common therapy target is by inhibiting γ-secretase; these types of drugs are collectively known as Gamma Secretase Inhibitors (GSIs). Nevertheless, blocking monoclonal antibodies (mAbs) is another strategy to inhibit Notch signaling. Figure were created with BioRender.com (accessed on 13 January 2022).

Finally, the last player in this pathway is the transcription factor that is continuously bound to the DNA called CSL. CSL is composed of CBF1/RBPJ-κ, suppressor of Hairless and LAG1 [12] (Figure 1).

The activation of the pathway is provoked by the ligand–receptor interaction between two proximal cells. When ligands interact with receptors, the NECD is cleaved by disintegrin and metalloproteinase (ADAM). The NICD of NOTCH is released by a γ-secretase/presenilins complex. This action allows NICD to enter the nucleus and displace co-repressors (CoRs) by binding itself to the transcription factor CSL, recruiting, at the same time, co-activators (CoAs). These events start the transcriptional activation of the target genes [11,13,14]. Some of those genes are in charge of cell cycle regulation, such as *MYCC* [15], *CCND1* [16], *CDKN1A* [17], and *BCL2* [18,19]; embryogenesis and stemness, such as the Hes family [20]; or genes related to proteins present in T-lymphocytes, such as CD25 [21].

## 3. Notch Signaling in Hematopoiesis

The important role of Notch in embryos was described by Robert-Moreno et al. when they studied *Notch1* mutation in mouse embryos. They showed that a mutation in this gene entails deficiencies in intra-embryonic hematopoiesis [22]. In adults, Notch has a crucial position in hematopoiesis: its inhibition can alter hematopoietic linages due to its importance in cell fate [23]. Indeed, *Notch1* has a major role in adult hematopoiesis by controlling events, such as lymphoid vs. myeloid differentiation [24], T vs. B lymphoid fate [25,26,27,28], αβ vs. γδ T-cell fate [29], and possibly CD4 vs. CD8 T-cell lineages [30,31]. Furthermore, Notch, certainly *Notch1* [32], is needed for the expansion of the hematopoietic stem cell (HSC) compartment [24].

On the other hand, other authors proved that Notch could be dispensable for adult HSCs. Maillard et al. inhibited the complete cascade of Notch signaling by two methods: firstly, by using dominant negative Mastermind-like1 (DNMAML), a potent and specific pan-inhibitor of Notch-mediated transcriptional activation; secondly, by silencing the *Rbpj* gene, through which canonical signaling converges from the different Notch receptors. The group examined the role of Notch in the HSC function in normal mouse bone marrow (BM) and in chimeras with an HSC transplant after radiotherapy. They observed a stable long-term reconstitution and a normal HSC engraftment either in a normal BM or a stressed BM environment. Therefore, they concluded that Notch signaling is not essential for HSC self-renewal. Nevertheless, they did not discard the fact that increased Notch expression could stimulate HSC self-renewal under other stress conditions different from radiotherapy [33]. Additionally, Notch target genes were expressed at low levels in primitive hematopoietic progenitors. Consequently, they postulate that levels of Notch in adult HSCs are too low to be translated into a detectable physiological function [33]. Similar conclusions were obtained by Duarte et al. ten years later, when they deleted *Rbpj-κ* by a *Vav-Cre* and *Mx1-Cre* system in adult BM [34].

## 4. The Role of Notch in Hematological Neoplasia

In this section, we briefly discuss the role of Notch in lymphoid malignancies, since this signaling pathway has been broadly study in this field; then, we examine Notch implications in myeloid neoplasia to finally focus on the alteration of this conserved pathway in AML.

Almost 60% of patients with T-cell acute lymphoblastic leukemia (T-ALL) have mutations of *NOTCH1* [35], but the prognosis of this mutation is not clear and seems to be dependent on additional genetic lesions [36,37,38]. The high ratio of mutations in this gene suggest that T-ALL is the hematological neoplasia most closely related to this signaling pathway. In this neoplasia, *NOTCH3* promotes *JAG1*, a phenomena that is caused by a *NOTCH3/JAG1* auto-sustaining loop [39]. This mechanism would produce a positive auto feedback and a stimulant paracrine effect in the adjacent cells. The loop seems to support the survival, proliferation, and invasion of leukemic cells and contributes to the development and progression of T-ALL [39]. Moreover, this signaling pathway could have prognostic value in those patients suffering from chronic lymphocytic leukemia (CLL). There are multiple studies that support *NOTCH1* mutations as a negative predictor factor of CLL patients [40,41,42]. Indeed, some authors found *NOTCH1* mutated in 11% of CLL patients and found that mutations in this gene are in 90% of the cases mutually exclusive with *TP53* disruptions, and confer a similarly dismal prognosis with a reduction in the overall survival (OS) [41]. Therefore, the role of Notch seems fundamental for lymphoid neoplasia.

In the myeloid linage, the forced expression of Notch increased progenitor differentiation toward the T lymphoid and megakaryocyte–erythroid progenitor (MEP) lineages. Consequently, the inhibition of Notch increased the granulocyte–monocyte progenitor (GMP) and produced a phenotype compatible with chronic myelomonocytic leukemia (CMML) in mice. Interestingly, in human CMML samples, there were some mutations that inactivated Notch-related genes, such as *NCSTN*, *APH1*, *MAML1*, and *NOTCH2*, which, to date, have not been described [43].

There are plenty of studies that seek to resolve the role of Notch in AML. Interestingly, *NOTCH1* is mutated in 12% of AML patients and its mutation is an adverse independent predictor factor for OS; nevertheless, the cohort analyzed was small (50 patients) and the results were not replicated, at least in part, due to the non-inclusion of Notch pathway genes in targeted NGS panels employed in myeloid malignancies [44]. In addition, one hundred de novo AML patients showed a greater gene expression of *NOTCH1*, *JAG1* and *DLL1* compared to normal donors, and a greater expression of these genes was an adverse predictor factor in the multivariate analysis [45]. A larger study performed on 363 samples and cell lines found that NOTCH1 and NOTCH2 were the receptors with the greatest gene expression in AML. Nonetheless, only NOTCH4 was significantly overexpressed when compared to healthy controls. Effectors of the Notch pathway, namely *HES1* and *DTX1*, showed a significant reduced expression compared to healthy controls, which suggest reduced Notch signaling in AML samples [46]. Additionally, the authors showed that the activation of *NOTCH1*, *NOTCH2*, and *HES1* led to reduced AML growth in vivo [46]. Likewise, Notch inhibition via dnMAML enhanced proliferation in vivo, thus revealing the inhibition of AML growth in response to Notch signaling [46]. More studies support the idea of Notch inhibition producing a greater proliferation; for example, Kang et al. showed that the reduction in Notch activity produced an increase in the pool of multipotent progenitors [47]. Furthermore, Lobry demonstrated that the Notch signaling pathway is inhibited in the whole leukemic cell population of human and mouse fractions of AML-initiating cells. They found increased levels of H3K27me3 in the promoter of different Notch target genes, which could explain their reduced expression. Lobry and his colleagues activated Notch by using a gain-of-function in vivo model. Notch activation induced cell cycle arrest, differentiation and apoptosis in the fraction of AML-initiating cells [48].

However, not all the studies defend the fact that the inhibition of Notch leads to an increase in the proliferation in AML. Different results were obtained by Li et al. when they studied the repercussion of the combination of tyrosine kinase inhibitors (TKIs) and the γ-secretase inhibitor (GSI) on FLT3-ITD + AML in vitro and in vivo. They found that the combination of these drugs reduced cell proliferation and induced apoptosis [49]. Similar results were obtained when experiments were performed on the HL60 cell line, in which high levels of *NOTCH2* and *JAG1* are shown. The inhibition of the Notch pathway, by using GSIs, blocked the cell-cycle progression during the G0/G1 phase and induced apoptosis [50]. These results are different from those previously explained, where Notch activation reduced cell cycle. However, in this study, Notch inhibition seems to reduce cell-cycle entry.

Finally, other studies do not focus on the role of Notch in the cell cycle of AML, but they study whether or not Notch has an anti-leukemic effect on xenograft models. The work of Kamga shows that mice treated with the γ-secretase inhibitor in combination with chemotherapy increased the overall survival, compared to the mice in which only chemotherapy was employed. For this reason, the authors suggested that Notch can be useful as a prognostic marker and therapeutic target in AML [51]. Therefore, since Notch seems important not only in hematological neoplasia, but in several tumors, some drugs were developed for its inhibition.

More than 50 clinical trials with drugs targeting Notch signaling in different tumors [52] were carried out. Nevertheless, it is remarkable that most of these trials do not focus on hematologic malignancies. Additionally, although there are two ways to inhibit Notch, by using blocking monoclonal antibodies (mAbs) and by inhibiting γ-secretase, the latter is the most well-known strategy, especially in hematological neoplasia.

Despite the efforts made to inhibit this pathway, most of the trials stayed in phase I with no results available (RO4929097), or with results but no further research (MK0752 and BMS-906024). Indeed, in the study of MK-0752, only a limited number of patients tolerated the proposed dose, while in the study of BMS-906024, 32% of patients showed at least a 50% reduction in bone marrow blasts [53,54]. On the other hand, LY3039478 reached phase II, but no objective responses were obtained (Table 1). In all these trials, Notch inhibition was employed in monotherapy, which could explain the lack of efficacy observed. In this review, we seek to clarify the role of Notch in the cell-cycle regulation of LSCs in order to be able to use Notch-targeted treatments to force LSCs to enter cell cycles. Therefore, an additional chemotherapeutic agent should be needed to eradicate the cycling leukemic population.

Only one trial reached the third phase of clinical development, the GSI called Nirogacestat (PF-03084014). Nevertheless, this approval is for desmoids tumors [55].

Even if we manage to properly inhibit Notch, the function of this pathway in AML is not only likely to be dependent on other genetic mutations, but also on the crosstalk with other signaling pathways.

## 5. Crosstalk and Non-Canonical Activation

There are multiples pathways that are related to Notch. Among them, we focus principally on Wnt and Hh, which seem to have a high grade of interdependence with Notch not only during embryo development, but also in stem cell regulation and the differentiation of different tissues.

Some authors explain how the accumulation of active β-catenin levels are negatively regulated by Notch at a post-translational level in stem and progenitor cells [56]. Other authors suggest that stromal *NOTCH2* induces a strong activation of Wnt signaling in chronic lymphocytic leukemia (CLL) tumoral cells [57]. Furthermore, it has been recently described that increasing Notch levels and decreasing Wnt activity leads to a cell-cycle arrest, delays disease progression, and increases overall survival in mouse models. Additionally, this therapeutic strategy would have no effects on normal HSCs [47]. Similarly, Li suggested that the inhibition of the Notch signaling pathway entails stem cell proliferation and hematopoietic cell regeneration. On the other hand, the inhibition of Wnt signaling pathways has the opposite effect [58]. In further experiments, Li et al. compared the effects in mice of inhibiting Notch versus inhibiting Notch and activating Wnt. The study showed that altering both pathways induces a greater stem cell proliferation and increases regeneration in sensory progenitor cells [59]. These results present the possibility of a strong crosstalk in cell-cycle regulation between both pathways.

Another important conserved pathway is Hh, as we explained in a previous work [60]. Nicolas shows that ablation of Notch1 results in epidermal and corneal hyperplasia, followed by the development of skin tumors through Gli2 upregulation [61]. In contrast, other authors explain that Notch induces the expression of *Shh* and *Hes3* [62]. Certainly, the correlation of Notch and Hh is also carried out by the regulation of *Hes1* by Hh, independent of canonical Notch signaling in stem-like cells [63]. The study of Domingo-Domenech shows how the inhibition of Notch and Hedgehog reduces the capacity to initialize prostate cancer [64]. The bona fide data of Hh and Notch relationship were obtained from a phase III clinical trial for Alzheimer’s disease. These patients were treated with a GSI to block β-amyloid production (GSI450139), and the incidence of non-melanoma skin cancers was 2% in the placebo cohort versus 10–11% in the GSI group [65]. This kind of neoplasm is related to Hh deregulation and this finding, obtained from clinical observations, shows the real and strong interconnection between Hh and Notch pathways.

More proof of the relationship between Hh, Wnt and Notch was obtained by Okuhashi et al. These authors found in in vitro experiments that *NOTCH1* was overexpressed when *GLI1* and *CTNNB1* were inhibited by a knockdown [66]. The interaction between these three signaling pathways was also described in a different type of myeloid neoplasia, such as chronic myeloid leukemia [67].

In this section, we summarized the important crosstalk between two signaling pathways, Hh and Wnt, which are also key players in embryo development. Nevertheless, there are several signaling pathways that can interact with Notch. To further explore the different crosstalk between Notch and other signaling pathways, we encourage the reader to read a specialized review about the topic. For example, Guo et al. described in their review HER/ErbB, PDGF/PDGFR, TGF-β, VEGF/VEGFR-2, IL-1, and IL-6 in breast cancer [68], but there are multiple manuscripts dealing with the crosstalk of JAK-STAT [69], MAPK [70,71,72], PI3/AKT [73], NF-κB pathway [74] or P53 [75].

## 6. Discussion

Since 1914, researchers tried to understand the function of Notch using different approaches. Nowadays, we know that Notch is one of the pathways in charge of the promotion of cell-cycle genes, such as *MYCC*, *CCND1*, *CDKN1A*, *BCL2*, and embryogenesis and stemness-related genes, such as the Hes family [15,16,17,18,19,20]. In this review, we addressed the role of Notch, physiologically and pathologically. The fact that Notch is a conserved pathway highlights its importance [6,7,8]. In embryogenesis, Notch has an essential role not only in embryo development, but also in the hematopoiesis during this period. In contrast, Notch seems dispensable for HSC maintenance in adulthood [33,34]. In spite of this apparent lack of importance in HSC maintenance, Notch seems to be a key player in cell fate determination. In fact, Notch controls lineage differentiation at different stages, such as lymphoid vs. myeloid cell differentiation or T vs. B lymphoid fate [24,25,26,27,28,29,30,31]. Taken together, all these observations highlight Notch as an important pathway in hematopoiesis.

Notch has been widely studied in lymphoid malignancies, where there is a broader body of evidence for its dysregulation, given the mutational frequency observed in these neoplasms. Nonetheless, we tried to focus on the role of Notch in AML. An interesting finding is the mutational frequency (12% of the patients, similar to CLL) of *NOTCH1* found in AML patients and the adverse outcome conferred either by mutations in this gene or the overexpression of different elements of this pathway [44]. Therefore, the Notch pathway seems to have a role in AML pathogenesis.

We sought to gather the available information regarding Notch in LSCs of AML and, more precisely, about its cell-cycle regulation. Information about this topic is contradictory and limited. Should we inhibit Notch to decrease the quiescent population of LSCs? Or is it contrariwise? To date, some studies demonstrated that the inhibition of Notch increased cell proliferation [46,47]. Lobry studied AML leukemia initiating cells (LICs) arriving at the same conclusion [48]. Therefore, it would be feasible to think about Notch inhibition as a therapeutic strategy; we would seek to force the LSC population to increase the cell cycle and, in this way, its chemosensitivity. Moreover, it is remarkable that the studies of Kannan and Lobry obtained similar results by two different approaches. Kannan studies AML cells as a whole, while Lobry studied AML-initiating cells, which are similar to LSCs [46,48]. By contrast, different studies performed in vitro and in vivo showed how the inhibition of Notch signaling pathways by the γ-secretase inhibitor reduces the cell cycle [49,50].

Since Notch could be dispensable in the maintenance of adult HSCs, it may be possible to inhibit this signaling, as previously suggested. Indeed, multiple clinical trials have been conducted, which aimed to inhibit Notch, mostly by using a γ-secretase inhibitor. Unfortunately, only one of those studies reached the phase III clinical trial in desmoids tumors. Nevertheless, the studies in hematological neoplasia did not progress from phase I and only LY3039478 reached phase II. We should not forget that most of the studies inhibit the γ-secretase protein, which is part of the canonical activation of the pathway. Therefore, although the full inhibition would be achieved, non-canonical activation caused by other pathways is possible. There is a large list of other players, but we focused on the two other pathways, Hh and Wnt, essential in embryogenesis. The evidence sustains the relationship between these pathways from in vitro, in vivo, and even clinical studies. We believe that the combined targeting of Notch, and the Hh and Wnt pathways can show synergistic effects in the quiescence reduction in LSCs and improve the clinical benefits over the targeting of a single pathway.

## 7. Conclusions

Notch may be dispensable for the maintenance of adult HSCs, which leaves an open door to target this signaling pathway. It is necessary to understand the role of Notch in leukemic stem cells to answer a fundamental question: does the inhibition of Notch reduce the quiescence state of LSCs by forcing cells into a cell cycle or enhance that quiescence state?

Current leukemic and oncologic treatments focus on the prevention of cell division to reduce the quantity of malignant cells in patients, but this approach does not preclude the possibility of a relapse. One of the most feasible methods to reduce this possibility would be by eradicating the cells that cause the relapse: the LSCs. The LSCs used to be in quiescence; therefore, conventional chemotherapy does not affect them.

In order to exploit the therapeutic potential of the Notch pathway, firstly, we believe it is essential to decipher the role of Notch in the regulation of the quiescence of the LSC population. Single cell technologies might help us in this difficult task. In a further step, targeted therapies could be employed, either directed only towards Notch or also directed towards Hh and/or Wnt, alongside conventional chemotherapy. This therapeutic strategy could decrease the quiescence of LSCs, increase their chemosensibility and achieve the eradication of LSCs and AML curation. Our group is currently working on this line of research and would be delighted to collaborate with other groups interested in this promising field.

## Figures and Tables

**Table 1:** Clinical trials for hematological malignancies. Source: https://www.clinicaltrials.gov/ (accessed on 10 February 2022). C: completed; T: terminated (ending prematurely); W: withdrawn.

Drug	Mechanism of Action	Clinical Trial	Phase	Status
RO4929097	γ-secretase inhibitor	NCT01158274	I	C
		NCT01088763	I	T
		NCT01236586	I	W
LY3039478	γ-secretase inhibitor	NCT02518113	I/II	C
MK0752	γ-secretase inhibitor	NCT00100152	I	T
BMS-906024	γ-secretase inhibitor	NCT01363817	I	C
Nirogacestat PF-03084014	γ-secretase inhibitor	NCT00878189	I	C
